# Socioeconomic differentials in fertility in South Korea

**DOI:** 10.4054/demres.2021.44.39

**Published:** 2021-05-11

**Authors:** Sojung Lim

**Affiliations:** 1 Department of Sociology and Anthropology, 0730 Old Main Hill, Utah State University, Logan, UT 84322, USA.

## Abstract

**BACKGROUND:**

South Korea has one of the lowest fertility rates in the world, reaching a record low of 0.98 in 2018. Understanding socioeconomic differentials in fertility in South Korea has become an important social and policy issue.

**OBJECTIVE:**

This study examines socioeconomic differentials in first and second childbirths among married women using various indicators of socioeconomic status at the individual and household level.

**METHODS:**

Using the Korean Labor and Income Panel Study (1998–2017), discrete-time hazard models are used to evaluate the relationships between multiple indicators of socioeconomic status and the transition to first and second births.

**RESULTS:**

Higher socioeconomic status (e.g., husband’s college education and standard employment, homeownership) is conducive to a transition to parenthood and second births. However, the wife’s employment – standard employment in particular – is negatively associated with both first and second childbirth. Among the indicators of socioeconomic resources, stable housing arrangements and the husband’s employment security appear to be the most important factors for a married couple’s fertility decisions.

**CONCLUSIONS:**

Socioeconomically disadvantaged married couples tend to delay their transition to parenthood. In addition, those with high SES are more likely than their counterparts with low SES to have second births. If these patterns persist, they have important implications for the demographic process and social stratification.

**CONTRIBUTION:**

The findings of this study contribute to a comprehensive understanding of socioeconomic differentials in fertility in South Korea and therefore have important policy implications. These findings will also prove useful to other societies with very low fertility rates.

## 1. Introduction

Notably, South Korea and some other East Asian countries have lowest-low fertility, which is a total fertility rate at or below 1.3 (Billari and Kohler 2004). In 2010 the total fertility rate (TFR) in South Korea was 1.23 and reached a record low of 0.98 in 2018 (Statistics Korea 2019). The deteriorating economic circumstances of young adults have been posited as a major contributor to such a drastic decline in fertility. For example, South Korea (hereafter referred to as Korea) experienced a substantial increase in precarious work (e.g., nonstandard jobs) after the financial crisis of the late 1990s (see e.g., Kim 2016; OECD 2015). The skyrocketing housing prices, especially in Seoul’s metropolitan areas where the majority of the population is concentrated, have also been identified as a major barrier to young people forming families (Lee 2017). The Korean media have portrayed young adults as *Sam-Po Sedae,* a term referring to a generation that gave up three things, i.e., romantic relationships (courtship), marriage, and childbearing (Jung 2012).

Extremely low fertility rates in combination with rapid population aging have been the subject of public and policy concerns. A growing body of literature explores various factors associated with fertility behaviors, from women’s labor force participation to fertility intentions to the availability of family policies (Kim 2014; Ma 2016; Son 2018). However, surprisingly, socioeconomic differentials in fertility are understudied in Korea and other East Asian countries (Raymo et al. 2015). Many previous studies have focused solely on a specific socioeconomic condition at the individual level (e.g., wife’s employment or education), ignoring the potential influence of the husband’s or household’s socioeconomic conditions on fertility decisions (e.g., Song, Ahn, and Lee 2018). Prior research is also limited in that it does not consider the possibility that the role of socioeconomic characteristics in fertility may differ by birth order (e.g., Ma 2014; Yoo 2014). As a consequence, we have segmented evidence on socioeconomic differentials in fertility in Korea.

Using 20 years of data from a panel survey (Korean Labor and Income Panel Study, KLIPS), this study aims to fill this gap by examining socioeconomic differentials in first and second childbirths among married women using various indicators of socioeconomic status. Specifically, this study evaluates how the socioeconomic conditions of both wife and husband (e.g., educational attainment, employment type) and of household (e.g., annual income and homeownership) are associated with the transition to first and second births. The findings of this study will shed light on the socioeconomic determinants of fertility in Korea by providing a more comprehensive picture of socioeconomic differentials in parity-specific fertility, which have important policy implications. These findings also have implications for other societies that are experiencing low fertility and growing socioeconomic differences in family behaviors (see e.g., McLanahan 2004).

## 2. Theoretical and empirical background

### 2.1 Theoretical explanations of low fertility

It is well documented that in the past several decades, fertility rates have declined in many developed countries below the replacement level (approximately 2.1 children born per woman). According to second demographic transition theory, changes in values and attitudes, such as individualization and secularism, are the main drivers of low fertility and related family changes in Europe and other Western societies (e.g., Lesthaeghe and Surkyn 1988). While this framework may prove useful in explaining fertility decline in Western societies, it is worth noting that such ideational changes as increasing individuation (over family) have not been observed in East Asia (Atoh 2001). For example, there is evidence for changing attitudes about women’s labor force participation, but the desire for marriage and children has been relatively stable (Raymo et al. 2015).

Other scholars have emphasized the importance of economic factors for understanding low fertility. With the increase in female education and labor force participation, the opportunity costs of childbearing also increase (e.g., Becker 1981). According to this model, women’s economic independence – such as better education, higher earnings, or full-time employment – has a negative association with fertility. In addition, some scholars argue that the timing of marriage is affected by one’s career development due to rising economic inequality and that women’s economic contribution becomes important for family formation during times of labor market uncertainty (e.g., Oppenheimer 1988a, 1988b; Oppenheimer, Kalymijn, and Lim 1997). Economic models of fertility have become more relevant in recent years as economic inequality has grown. In fact, deteriorating economic conditions among young adults, particularly among young men (relative to women, who have experienced advancements in education and employment), have been attributed to socioeconomic differentials in union formation and childbearing in the United States (Cherlin 2014; Ruggles 2015) and Europe (e.g., Karabchuk 2020).

Economic explanations of low fertility have gained importance in East Asian countries, especially after the Asian financial crisis in the late 1990s. Korea and other East Asian societies have since undergone substantial changes in economic structure, resulting in the growth of nonstandard jobs (e.g., part-time and temporary work) and widening income inequality. Moreover, rising economic inequality and employment insecurity have created feelings of instability and apprehension, leading to greater investment in education and careers among young adults (e.g., Hobcraft 1996). Consequently, young people – in particular those who have limited career prospects – likely delay or even abandon family formation (Blossfeld and Hofmeister 2006; Karabchuk 2020). Such perceptions of economic insecurity, combined with rising housing prices and the increasing cost of children’s education, may have contributed to persistently low fertility in Korea and other East Asian countries (Anderson and Kohler 2013; Chen 2013; Kim 2013; Lee 2017).

While these theoretical explanations provide a generalized framework for understanding the socioeconomic determinants of family formation, it is also important to note that decisions about childbearing are made in a broad socioeconomic and cultural context (Rindfuss, Guzzo, and Morgan 2003). For instance, women’s fertility behaviors are largely affected by their ability to balance work and child-rearing responsibilities, which are further determined by country-specific institutions that include education, family, labor market, policy, and gender relations. Gender equity theory (McDonald 2000) hypothesizes that conflicts in terms of gender equity between individual-oriented institutions (e.g., education and the labor market) and family-oriented institutions causes a decline in fertility. It argues that a fertility decline to replacement level occurs with an increase in gender equity within the institution of the family, and that further decline to very low fertility (below replacement level) is associated with a mismatch in the level of gender equity between individual institutions and family-oriented institutions (McDonald 2000). By focusing on changes among various institutions, this hypothesis may help explain why fertility in East Asia is at its lowest, since tensions between changing opportunities for women (e.g., education and career opportunities) and persistent gender and family norms (e.g., rigid gender division of labor) may push many women to delay or forgo childbearing. Also, it should be noted that the labor market in Korea (and Japan) is still gender segregated, and career opportunities are often limited for women (Brinton 2001). This implies that the labor market in Korea is not a fully individual-oriented institution in that its structure is affected by and reflects traditional gender norms emphasizing women’s domestic role.

### 2.2 South Korean context

**Figure 1:**
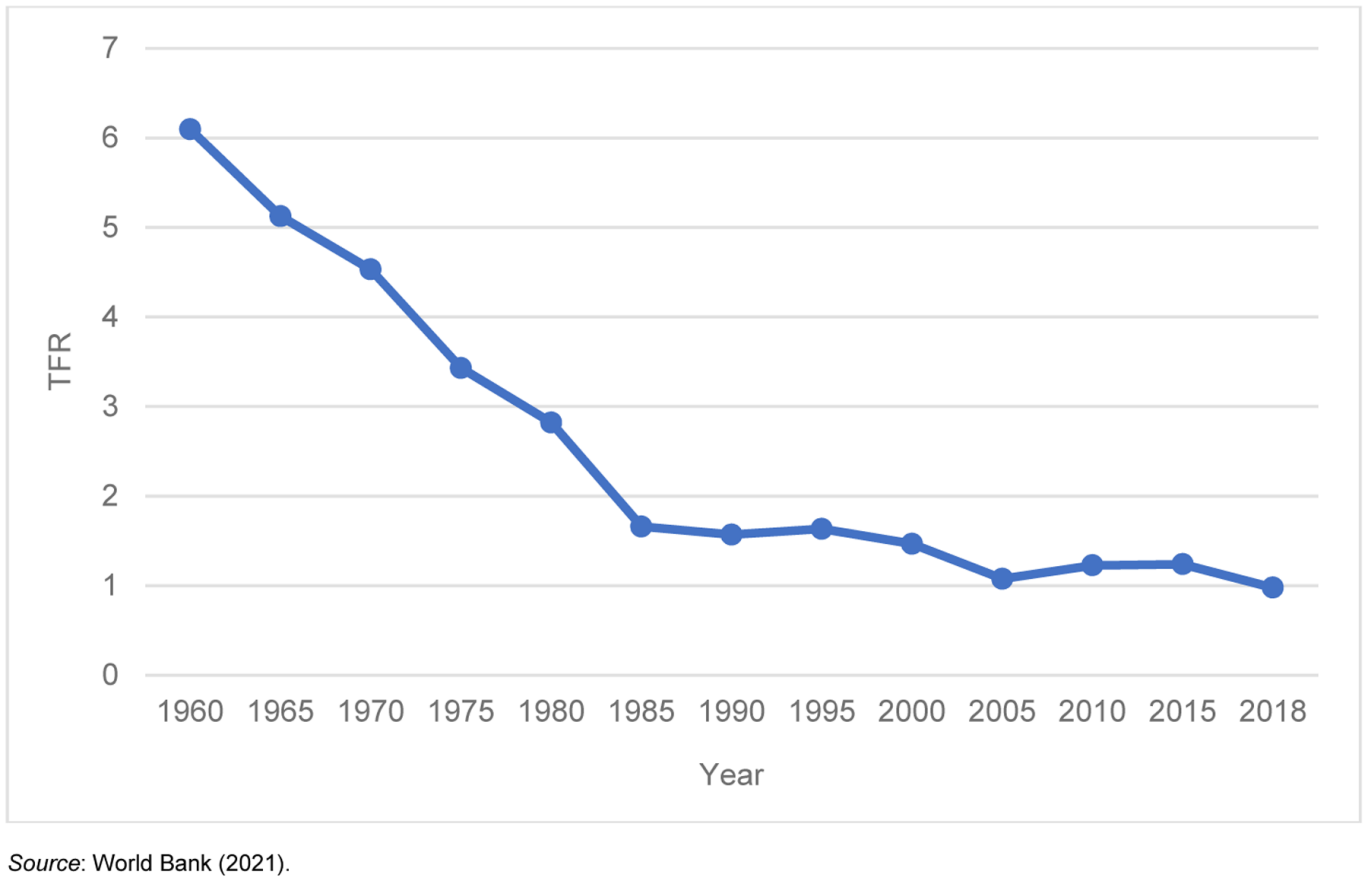
Total fertility rate in South Korea, 1960–2018 *Source*: World Bank (2021).

Figure 1 shows changes in the total fertility rate in Korea during the past several decades. Korea entered the low fertility regime in the mid-1980s, and its fertility rate has kept declining. In 2018 the TFR in Korea was recorded as 0.98, making Korea the only OECD country with a fertility rate below 1 (Statistics Korea 2019).

The fertility decline in Korea is largely driven by the trend toward delaying and even foregoing marriage, as childbearing occurs almost exclusively within marital union. As is well documented, the level of nonmarital fertility has been very low in Korea. In 2014 the percentage of nonmarital births was 1.9, the lowest among OECD countries, followed by Japan (2.3%) and Turkey (2.8%) (OECD 2014). The OECD average for nonmarital births was about 40%. Negligibly low levels of nonmarital childbearing are also attributable to the low prevalence of cohabitation in Korea: Koreans have conservative attitudes toward cohabitation, even compared to other East Asian countries (Eun and Lee 2005; Raymo et al. 2015).^2^

Reflecting the trend toward fewer and later marriages in Korea, the percentage of ever-married women aged 25–29 decreased from 83% in 1985 to 41% in 2005 (Kosis 2016). Similarly, the percentage of ever-married men aged 30–34 was 59% in 2005 with a corresponding figure of 93% in 1970 (Kosis 2016). In addition, in 2018 the mean age of women at first birth was 31.9 and the mean age at second birth was 33.6. It is also interesting that while most births occur among women between the ages of 30 and 34, women in their early 40s (ages 40 and 44) were the only age group with an increase in fertility in 2018 (Statistics Korea 2019).

As discussed above, deteriorating economic conditions for the younger generations have been linked to a continuous decline in fertility in East Asia (Raymo et al. 2015). Following the financial crisis of 1997, labor market segmentation between standard workers (*Jeong-gyu-jig*) and nonstandard workers (*Bi-jeong-gyu-jig*) has become salient in Korea. According to recent data, about one in three waged workers is classified as holding a nonstandard job, and the share of self-employed workers is over 20% (Ministry of Employment and Labor 2017). The percentage of nonstandard workers, including those in both waged work and self-employment, is unusually high in Korea compared to other OECD countries (OECD 2015).^3^ Nonstandard workers receive inferior treatment compared to standard workers in terms of wages, fringe benefits, and legal and union protection (e.g., Houseman and Osawa 2003; Kalleberg, Reskin, and Hudson 2000; Sohn 2011). More importantly, most nonstandard workers lack the mobility to move to standard work positions in the Korean labor market. Therefore, in the Korean context, employment type (i.e., standard vs. nonstandard job) is one of the key indicators of socioeconomic status, since it signals both current labor market status and future career trajectory and economic prospects (e.g., Kim 2016).

With rising inequality in the labor market the perception of economic insecurity and inequality has spread, in particular among young people. For example, *Sampo sedae* or *Opo sedae* have been popular media terms to describe the socioeconomic conditions of young adults. *Sam* means three, *o* five, *po* give up, and *sedae* a generation. Therefore, *Sampo sedae* refers to a generation giving up three things (dating, marriage, and children), and *Opo sedae* is a generation giving up five things (the first three plus employment and homeownership) (e.g., Jung 2012). According to a recent Global Attitudes Survey, Korean millennials are less likely than their counterparts in Europe and the United States to believe in meritocracy (e.g., education and hard work are very important to getting ahead in life) and in a better financial future for their children (Parker 2015). The skepticism of Korean young adults is puzzling, considering the country’s strong economy (e.g., Korea’s GDP was ranked 14^th^ in 2014) and educational achievements (e.g., Korean students notably outperform most advanced countries in math, science, and reading). Nevertheless, it is clear that Korean young adults are keenly aware of economic inequality and insecurity as major barriers to family formation.

It is also important to consider distinctive gender contexts in order to understand fertility behaviors in Korea. Korea and other East Asian societies are still influenced by traditional family values and gender norms in spite of the rapid expansion in female education and labor force participation (Bumpass and Choe 2004). In East Asian societies the traditional Confucian respect for learning and a competitive educational system emphasize women’s role in their children’s education (e.g., Hirao 2001). The responsibility of mothers for their children’s educational success has become even more emphasized in recent years as the prospect of regular, full-time employment has worsened for young people. Higher education for women in Korea has not always been linked to a stronger attachment to the labor force, since a wife’s education has been often viewed as an important asset that contributes to the husband’s high status (e.g., Lee 2001). This cultural and gender-specific expectation may complicate the picture of socioeconomic differences in fertility.

### 2.3 Socioeconomic status and family formation in South Korea

One important trend documented in recent decades is the widening socioeconomic inequality in family outcomes (e.g., Cherlin 2014; McLanahan 2004; Ruggles 2015). For instance, a growing body of literature documents the marital divide by educational attainment, wealth, and employment characteristics including employment type, fringe benefits, and union membership (e.g., Lim 2017; Schneider 2011; Schneider and Reich 2014; Schneider, Harknett, and Stimpson 2019). Observing a divergence in family structure and behaviors according to socioeconomic status, scholars argue that marriage has become a symbol of achievement and social status (Cherlin 2004) and that individuals of low socioeconomic status may not be able to meet the economic standards for marriage and raise children within a marital union (Edin and Kefalas 2011). In a context of labor market uncertainty and polarization, increasing economic inequality has been hypothesized to be the primary underlying cause of these changes (Cherlin 2014; Oppenheimer 1988a, 1988b; Ruggles 2015).

Whether a similar pattern can be observed in Korea and other East Asian countries is an interesting and important question. In particular, given the very low level of fertility in this region, whether individuals’ fertility decisions differ across socioeconomic strata has very important societal and policy implications. However, evidence from prior research on socioeconomic differentials in fertility is still limited. Existing literature also brings mixed results in that the relationship between socioeconomic status and fertility often varies by gender as well as by a specific indicator examined. For men, evidence suggests that higher socioeconomic status, such as income, education, or employment status (while differences in employment type are often ignored) is positively associated with parenthood, reflecting a strong expectation for men to assume the role of breadwinner (e.g., Ma 2016; also see Raymo et al. 2015 for a review). However, research on the role of socioeconomic conditions in fertility among women documents inconsistent evidence. Women’s labor force participation is negatively associated with childbirths, regardless of birth order (e.g., Han and Lee 2015). Another study finds that women’s employment after the first birth lowers the odds of having a second child (e.g., Ma 2016), but there is also evidence that women’s labor force participation is not associated with first births (Kim 2014).

In most developed countries, women’s education is closely linked to labor force participation (Goldin 1994). However, in spite of educational expansion among Korean women, the relationship between women’s education and employment has been found to be relatively weak. The pattern of Korean female labor force participation is still characterized by an M-shape, due to labor force withdrawal during prime ages of childbearing and early childrearing (e.g., Brinton and Lee 2001). Among all OECD countries, this pattern is only observed in Korea (OECD 2017). Whereas aggregate-level association between women’s education and employment and the pattern of female labor force participation over the life course has changed little, there is some evidence suggesting that the economic foundation of marriage might have been shifting. Recent research documents that more young Korean men prefer their wives to be continuously employed after marriage (Park and Lee 2017), and that marriage rates of the lowest-educated women, who have low economic potential and employment prospects, have rapidly declined (Park, Lee, and Jo 2013). There is also evidence that highly educated women are more likely to continue working after childbirth than their lower-educated counterparts (Ma 2014). Similarly, in a recent cohort in Japan, a country with a similar gender and labor market context to Korea, the effects of women’s earnings in marriage have become positive (Fukuda 2013).

At the same time, it is important to recognize that the educational differences in attitudes toward and behaviors surrounding childbirth found in Korea often contradict those found in other Western countries. Studies show that among unmarried Korean women, the highly educated hold more favorable views of marriage than those with lower education (e.g., Raymo and Park 2020). This finding may be related to a strong educational homogamy in marriage in Korea. In fact, Korea has the strongest educational homogamy in the world (Smits, Ultee, and Lammers 1998), and the proportion of married couples who are educationally homogenous has increased over time, reaching almost 80% in 2015 due mainly to an expansion of female education and the resultant resemblance in educational distribution across gender (Shin, Lee, and Park 2017). Prior research documents that educational differences in completed fertility are not large in Korea (Yoo 2014), but that differences may be pronounced regarding the decision to have second births as highly educated women delay or forsake having additional children because of incompatibility between work and family responsibilities (Brinton and Oh 2019).

Another important factor that may affect family formation is high housing costs. Due to skyrocketing house values, especially in Seoul and surrounding satellite cities, securing stable housing is a big concern for young adults. One common housing arrangement for those who do not own a home in Korea is called *Jeonse. Jeonse* is a real estate contract unique to Korea in which a renter makes a lump-sum deposit to a landlord, called ‘key money,’ when a lease is signed. The amount of ‘key money’ usually ranges between 50% and 80% of the property’s value. The *Jeonse* contract is usually made for a 2-year term and, once signed, the tenant stays at the property without paying any additional monthly payments as the landlord collects interest earned on the deposit during the contract period. The amount of key money has been increasing following rising house prices in recent years, and according to a report from the early 2010s the average key money to rent an apartment with *Jeonse* in Seoul has reached around 300,000 USD (Phillips 2014). Research shows that housing prices and the amount of key money for *Jeonse* contracts at the local level are negatively associated with marriage and birth rates (Lee 2017). For people who cannot secure seed money (through a mortgage or help from their families) to purchase a house or find *Jeonse, Weolse* (monthly rent) becomes a common housing arrangement, in particular among households headed by young adults (e.g., in their 30s) (Seoul Metropolitan Government 2018).

While limited, evidence from prior research arrives at two scenarios regarding the relationship between socioeconomic status and fertility in the South Korean socioeconomic context. If having a child is a normative pathway after marriage, but higher-order births are perceived to be optional since the married couple have met normative and individual expectations, then socioeconomic differentials might be more pronounced for second births than for first births. However, it is also plausible that few socioeconomic differences are found by birth order if young people in Korea delay (or give up) childbearing for economic reasons, in particular factors beyond individual control (e.g., lack of standard employment opportunities and affordable housing). In addition, there might be gender differences in the role of socioeconomic status in childbirths depending on a specific indicator examined in light of distinctive gender contexts in family and at work. In particular, women’s employment would be negatively associated with childbirths due to the difficulty of combining work and family responsibilities. On the contrary, husbands having a better labor market status (e.g., holding standard employment) would be conducive to the transition to childbirths, while having an insecure labor market status, especially a nonstandard job, would deter the transition, given the rigid labor market segmentation and the persistent male-breadwinner norm.

## 3. Data and methods

### 3.1 Data

Data come from the Korean Labor and Income Panel Survey (KLIPS). The KLIPS is an annual longitudinal study based on a representative sample of 5,000 households in urban areas at the baseline (1998). Through direct face-to-face interviews, the household reference person is asked to provide information on all household members. If face-to-face interviews are not available, telephone interviews and self-administered questionnaires are used. The analytic sample includes data from wave 1 (1998) to wave 20 (2017), the most recent survey available at the time of conducting the current analyses. The KLIPS well suits the purpose of this study since it covers the period from after the financial crisis of 1997 to the present, during which time economic inequality has increased in Korea.

In light of very low nonmarital birth rates in South Korea, only marital fertility is considered by restricting analyses to those in first marital union. This study examines (1) transitions to first births among childless married women and (2) transitions to second births among those with one child. Due to very low fertility and the possibility of selection (e.g., women with more than three children may differ from those with fewer children), any higher order births (e.g., third births) are excluded from the analyses (see Methods section for details).

The analytic sample includes women aged 20 to 49, 20 being the legal age for marriage without parental consent and fertility at older ages (over 40) having increased in recent years in Korea (Statistics Korea 2019). In addition, listwise deletion is used to handle missing cases: the range of missing cases of covariates included in the analyses is between 0% (e.g., ages of wife and husband, region, wife’s education and employment type) and 3.9% (husband’s education). The variables with most missing values are annual household income (7.9% for the first-birth sample and 2.3% for the second-birth sample) and husband’s employment type (ranging from 5.7% for the second-birth sample to 10% for the first-birth sample).^4^ After applying these selection criteria and listwise deletion, the final analytic sample comprises 2,297 person-year observations for the analytic sample for first births and 7,938 for the second-birth sample.

### 3.2 Measures

#### First and second birth:

The first birth is coded as 1 if women in their first marriage who were childless at survey year (t) had given birth by survey year (t+1), and 0 for women who were still childless at year (t+1). The second birth is coded as 1 if women in their first marriage with one child at survey year (t) had another childbirth by survey year (t+1), and 0 for women who still had only one child at year (t+1). This means that only childless married women at year (t) are included in the models estimating the associations between socioeconomic status and the first birth, since they can only have the risk of a first childbirth. Similarly, in models for the second birth, only those who had one child are included as they are exposed to the risk of a second birth. Married women are censored after the event of interest (i.e., first or second birth) occurs and those who do not make the transition (i.e., stay childless for the first birth sample or still have one child for the second birth sample) are censored at last interview or observation.

#### Socioeconomic status:

Various indicators of socioeconomic status at the individual and household level are considered in this study. First, educational attainment and employment type for both wife and husband are included: the level of education is a categorical variable, which identifies less than high school education, high school education (reference), some college or junior college degree, and university (or postgraduate) degree. The wife’s and husband’s employment type, based on information on labor force participation (e.g., being employed or out of the labor force) and employment status (e.g., regular, temporary, day labor, self-employment), is categorized as standard employment (reference), nonstandard employment (e.g., temporary work, day labor), self-employment (including family business), and non-employment. Following the classification of the Korean Statistics Bureau (see footnote 3) and considering the large proportion of self-employed in Korea, self-employment is included as a separate category in the analyses. Non-employment refers to those who are unemployed or out of the labor force.

Annual household income for the past calendar year (logged) is included as a proxy for family economic conditions. Household income (in 1 million KRW, approximately 830 USD) includes wages (from employment), capital gains, social insurance, income from real estate property, and income from all other sources. Homeownership is a categorical variable consisting of four housing arrangements: homeowner (reference), *Jeonse* (key money deposit), *Weolse* (rent with a monthly payment), and other types (e.g., coresidence with parents).

#### Controls:

All models control for demographic characteristics that might confound the relationship between socioeconomic status and fertility, including age of husband and wife and place of residence (Seoul (reference), metropolitan cities other than Seoul, and other areas). In addition, time since first marriage in the first-birth sample and time since first birth in the second-birth sample are included as a baseline hazard (see Methods section for details). The models for second births include an additional control variable, viz. the sex of the first child, as the decision to have a second child might be associated with the sex of the first child (Chung and Das Gupta 2007).

### 3.3 Methods

Since the relationship between various indicators of socioeconomic status and fertility may differ by birth order, models are estimated separately by parity (e.g., Brinton and Oh 2019; Kim 2014). Discrete-time hazard models are employed for predicting the transition to first and second birth as a function of socioeconomic status, given the outcome of interest (i.e., had a childbirth in a given year) and the nature of the data (i.e., annual survey). Discrete-time hazard models are useful in that they consider the role of duration (baseline hazard) while examining the extent to which women’s transition to motherhood varies in relation to their socioeconomic status. Baseline hazard of birth is specified using duration since first marriage for the first-birth sample and duration since first birth for the second-birth sample. Specifically, time (duration) since first marriage/birth is defined by splines of duration comprising 0–1 year (since first marriage/birth), 2–3 years, 4–5 years, and 6 or more years, based on the results of preliminary analyses.^5^ All variables included in the analyses, except for the sex of the first child, are time varying and are lagged by a year so that socioeconomic and other demographic characteristics precede the event of birth (e.g., Schneider 2011).

## 4. Descriptive results

Table 1 presents sample characteristics (means/percentages and standard deviations) by birth order. During the observation period, 22.8% of women give birth for the first time and 12.7% of women (who had one child) have second births between two waves. The mean age at first childbirth is 29.4 years for women and 31.6 years for husbands and the corresponding figures for a second childbirth are 30.5 and 33.0 (results not shown). The mean duration since first marriage for those in the first-birth sample is 3.55 years and the mean duration since first birth for those in the second-birth sample is 7.08 years.

Turning to individual socioeconomic characteristics, a substantial proportion of women are out of the labor force, 40.8% for the first-birth sample and 56.3% for the second-birth sample, consistent with the relatively low rates of labor force participation for married women in Korea (Brinton and Lee 2001). Among those who are employed, husbands are much more likely than wives to work in standard employment. In addition, the percentage of university graduates is higher for husbands than wives, reflecting the tendency for educational hypergamy/homogamy in Korean society.

More importantly, Table 1 reveals interesting differences in socioeconomic characteristics by birth order. More than half of women in the second-birth sample are not working, which is 15% higher than for the first-birth sample. This indicates that a substantial proportion of women who were still working after marriage left work after their first childbirth. Similarly, the proportion of women in regular, standard jobs declined by 11% in the second-birth sample. These findings show that women’s labor force participation is still largely affected by childbirth (Brinton and Lee 2001; Ma 2014). However, the distribution of employment status among husbands is almost identical in the first-birth and second-birth samples. At the same time, educational differentials become more pronounced for those in the second-birth sample, in that the proportion of those without a high school degree declines for both wives and husbands. Also, the distribution of homeownership shows that those in the second-birth sample are more likely to own their homes (48.3%) than those in the first-birth sample (38.8%). Considering that only 13.2% of Koreans in their 30s (24.3% in 40s) reported owning homes in 2017 (Statistics Korea 2018), it appears that homeownership might be conducive to the transition to parenthood (and potential subsequent births).

## 5. Multivariate results

### 5.1 Results for the transition to first birth

Tables 2 and 3 present results from discrete-time hazard models predicting the likelihood of having the first/second birth as a function of various indicators of socioeconomic status.^6^ Considering the nature of the outcome variable of interest (i.e., whether a woman had a first/second birth between two waves), logistic regression models were used. Results are presented with coefficients and 95% confidence intervals to show the uncertainty of results (Bijak 2019). In addition, figures showing the predicted probabilities of a first and second birth by a specific indicator of SES across duration (0–1 year, 2–3 years, 4–5 years, and more than 6 years since first marriage/birth) are presented in the Appendix. Predicted probabilities are calculated from models that include control variables and duration along with the indicator of interest (e.g., wife’s educational level). That is, the figures illustrate probabilities of women having their first or second birth at each duration while holding all covariates at their mean across different levels of socioeconomic status.^7^

Results from Model 1 in Table 2 show that the risk of a first birth is highest within the first 23 months of marriage and then declines with marital duration (see also Appendix A). The likelihood of having a first birth drops a great deal after 6 years of marriage, reflecting the relatively short period of time between marriage and first childbirth in Korea. In addition, women with tertiary education (i.e., junior college or university degree) have a higher risk of first birth than high school graduates. However, women’s lower education (i.e., no high school degree) is negatively associated with the transition to parenthood. According to Model 2, which examines the role of husband’s education in first birth, a college education increases the risk of first birth. Also, women with lower-educated husbands (no high school degree) are less likely to have a first birth than those whose husbands have a high school education, but it should be noted that the 95% confidence interval includes zero (−1.438 to 0.153).

Turning to relationships between wife’s employment type and the likelihood of first birth (Model 3), wives who are out of the labor force are more likely to have first births than employed women (in both standard and nonstandard employment). Those in self-employment also have a lower risk of first birth than non-employed women, but the 95% CI includes zero (−0.869 to 0.001). It is likely that women who plan to have a child may have left the labor force for that specific purpose, especially those whose husbands have better economic standing. In fact, women tend to be out of the labor force if their husbands hold standard employment (supplementary analyses, results not shown). It is also worth noting that wives’ employment type makes little difference to the likelihood of them having a first birth. Even those with careers in standard employment who may benefit from recent policy changes (e.g., parental leave) do not differ from wives in unstable nonstandard jobs in terms of the likelihood of having a first birth. In addition, husbands’ non-employment deters the transition to parenthood, according to results from Model 4. This finding is not surprising, in light of the normative expectations for men to be breadwinners in Korean society. Both standard work and self-employment are positively associated with first birth, but the 95% confidence interval for nonstandard employment includes zero (−0.026 to 1.504).

The next two models show results for household-level socioeconomic resources. According to Model 5, additional household income (of 1,000,000 KRW, which is approximately 850 USD, converted to log) is not associated with first birth. However, the likelihood of first birth is associated with type of housing arrangement (Model 6): those with *Weolse* (monthly rent) are less likely to have a first birth than homeowners, suggesting the importance of a stable housing arrangement to the timing of family formation (e.g., Chen 2013; Lee 2017). However, the coefficients for other arrangements (e.g., coresidence with parents) and *Jeonse* (key money deposit) do not differ from zero.

Model 7 includes all posited SES indicators in order to evaluate whether the associations observed in Models 1–6 change with the inclusion of all indicators. First, the strong positive association between tertiary education of the wife and the risk of first birth observed in Model 1 is attenuated. However, the disadvantage of lowest-educated women (relative to high school graduates) is still observed. In addition, the husband’s educational level is no longer associated with first birth when the wife’s education is considered (supplementary analyses, results not shown). On the contrary, the relationship between employment type of both wife and husband and first birth (Models 3 and 4) remains the same: the wife’s employment delays the transition to first birth while the husband’s employment facilitates the transition. In addition, the coefficient for the husband’s nonstandard employment is now different from zero (95% CI are 0.124 to 1.730). Annual household income is not associated with the likelihood of first birth, as was found in Model 5. At the same time, the negative association between *Weolse* (monthly rent) and first birth is attenuated: the 95% confidence interval now includes zero (−0.853 to 0.015).

### 5.2 Results for the transition to second birth

Next, Table 3 presents results for the transition to second childbirth. According to Model 1, the risk of a second birth increases with the onset of exposure (i.e., first birth) and reaches its peak 2 to 3 years after having the first child. After that, the likelihood of having a second child declines (also see Appendix B).

Results from Model 1 also show that wife’s education is not associated with the transition to second birth. Lowest-educated women (no high school degree) have a lower likelihood of a second birth than high school graduates, but the 95 CI includes zero (−1.088 to 0.023). The results for husband’s education (Model 2) are similar for first births (Table 2), as women with college-educated husbands are more likely to have second childbirths than other groups. In addition, results from Model 3 show that the wife’s employment status is not related to the likelihood of having a second childbirth, which stands in contrast to the strong negative association between the wife’s labor force participation and first birth (Model 3, Table 2). It is worth noting that the coefficient for the wife’s standard employment is negative, although the 95% confidence interval includes zero (−0.324 to 0.018). As for the husband’s employment type (Model 4), standard employment facilitates the transition to second birth. However, women whose husbands have nonstandard employment do not differ from those whose husbands are not employed in terms of the likelihood of a second birth. At the same time, husband’s self-employment is positively associated with a second birth, but this should be interpreted with caution as the 95% CI includes zero (−0.034 to 0.763). These results suggest a bifurcation in second births between husbands in standard employment (and self-employment) and husbands in nonstandard/non-employment. Together with the results for first births, it seems that married couples may delay having a second child, or give up altogether if the husband’s employment status is unstable, once they have satisfied the normative expectations of parenthood. With regard to socioeconomic conditions at the household level, household income is positively associated with the risk of a second birth (Model 5): an additional 1,000,000 KRW (converted to log) increases the odds of a second birth by 13% (exp(0.123) = 1.131). In addition, housing arrangements still play an important role in second births (Model 6). Those with *Weolse* (monthly rent) have the lowest likelihood of having a second birth, while no differences are found among other groups.

In Model 7, which includes all indicators of socioeconomic status, most indicators are not associated with second childbirths. One exception is that the negative association between women’s standard employment and second births (Model 3) becomes stronger: women in standard employment are least likely to make the transition to a second birth among women of various employment statuses.

## 6. Conclusions and discussion

As one of the countries with the lowest-low fertility and fastest-aging population, the issue of fertility decline has become a subject of serious concern in Korea. The government’s efforts to reverse the falling fertility trend include subsidizing childcare leave, child allowances, and expansion of nurseries, but with little success. Given the level of societal attention and the governmental resources provided to address this issue, it is surprising that we still have a limited understanding of fertility behaviors across socioeconomic status. To fill this gap in the literature, the present study examines the socioeconomic characteristics of both wives and husbands and household economic conditions, and their relation to transitions to first and second childbirth.

Results from discrete-time hazard models provide evidence of substantial socioeconomic differentials in fertility. For instance, the husband’s higher education and better employment status (e.g., college education, standard employment) are positively associated with having a first and second childbirth. Also, those who can secure enough money to purchase a house or to deposit a lump-sum for a *Jeonse* lease have a higher likelihood of having first and second births than those who rent by the month (*Weolse*).^8^ At the same time, it is important to note that the association between socioeconomic status and fertility often varies across indicators and gender. In general, higher socioeconomic status seems to be conducive to the transition to parenthood and second birth; however, employed wives are less likely than housewives to have a first child. This reflects the difficulty of combining work and motherhood due to gender norms emphasizing women’s domestic responsibilities and a family-unfriendly work culture (e.g., long work hours and after-work social gatherings) (Brinton 2001; Choe, Bumpass, and Tsuya 2004). While women’s labor force participation is negatively associated with childbirths, the underlying reasons for delaying births may differ by employment status. For example, women in nonstandard employment, who in light of a strong preference for status homogamy in Korea are likely to have husbands with insecure jobs, might delay (or give up) childbearing for economic reasons. On the other hand, women with standard jobs might postpone childbearing for career reasons, since the opportunity costs of leaving standard employment are high in the Korean labor market, where it is almost impossible for married women to find standard employment upon reentry (Brinton 2001). Indeed, the likelihood of making the transition to a second birth is the lowest among wives in standard employment (Model 7, Table 3). A recent qualitative study on highly educated Korean women provides supporting evidence for this possibility by documenting that those who continue working full-time consider having only one child (Brinton and Oh 2019).

In addition, fertility differentials by husbands’ employment status are worth taking into consideration. Women have a lower likelihood of giving birth if their husbands hold nonstandard jobs compared to those whose husbands work in standard employment or self-employment. As discussed above, employment status is not only an indicator of one’s current labor market status but also of one’s future career trajectory in a rigidly segmented Korean labor market. In this context, married couples may postpone having their first child or subsequent children if the husband is not in secure standard employment (or does not run his own business), reflecting the persistent male-breadwinner norm. These findings are consistent with the finding that deteriorating employment prospects for young men are linked to declining marriage and fertility rates (Cherlin 2014; Karabchuk 2020; Ruggles 2015; Yeung and Yang 2020). By considering employment types of both husband and wife, this study also finds that the role of employment in fertility may differ depending on whose labor force participation is examined. Given that prior studies on fertility have often focused only on one spouse’s employment (e.g., including wife’s employment status without considering the effect of husband’s labor market status) (e.g., Kim 2014; Ma 2016), the results of this study show that it is important to understand fertility decisions by looking at the dynamics of couples’ employment status.

Another important finding of this study is the role of homeownership in fertility behaviors. For both first and second births, those with *Weolse* (monthly rent) are least likely to make the transition to parenthood.^9^ With skyrocketing home prices and the lack of affordable housing, securing ‘key money’ becomes a challenge and is causing rising household debt in Korea (Sohn 2019). Therefore, policies to help relieve the burden of high housing costs for young couples need to be implemented and expanded as a measure to boost fertility. For instance, Seoul Metropolitan Government provides newlyweds with low mortgage rates and public housing (Seoul Metropolitan Government 2020).

Considering that the level of nonmarital fertility is negligible and that most married couples in Korea make the transition to parenthood within a relatively short period of time (Statistics Korea 2019), very low fertility can be attributable to a decline in marriage as well as a decline in the number of children born to married couples. The findings of this study show that socioeconomically disadvantaged married couples tend to delay their transition to parenthood. In addition, among married couples who have a first child, those with high SES are more likely than their counterparts with low SES to have second- (and probably higher) order births. If these patterns persist, they have important implications for the demographic process and social stratification. Therefore, policies to address low fertility should pay attention to the challenges faced by individuals of different socioeconomic status. For women who want to continue their career jobs (standard employment), for instance, policies to help lower the burden of balancing work and childcare, such as encouraging employers to create on-site childcare facilities through tax subsidies and increasing preschool and afterschool programs to provide extended childcare hours (e.g., early morning/evening, weekends), will encourage couples to have children. In addition to these policies, providing direct economic benefits (e.g., cash allowances upon birth) and ensuring employment stability (e.g., expanding parental leave to nonstandard workers and self-employees) may facilitate married couples with low socioeconomic status to make the transition to first and subsequent births.

This study is not without limitations. First, individuals with different characteristics (e.g., personality), which may be related to their socioeconomic status, can be selected into parenthood or into remaining childless. Employing analytic designs to explicitly deal with the issue of selection would be a useful extension in order to establish a causal relationship between socioeconomic status and fertility decisions. Second, building upon the findings of the current study, future research will need to examine socioeconomic differentials in fertility in more depth. For example, it is important to understand the role of housing debt and the source of money for housing purchases or *Jeonse* (key-money deposit) in fertility so as to design effective policy measures to reduce socioeconomic differentials in fertility and increase fertility rates. Also, gender division of labor might affect the transition to parenthood in light of the difficulty of combining work and family responsibilities that Korean women experience (Brinton and Oh 2019; Choe, Bumpass, and Tsuya 2004). The present study cannot examine the effect of gender equity on fertility as the KLIPS only collects information on hours of household work in supplementary surveys on time use (2004 and 2014). It is necessary for future research to evaluate how gender division of labor within the family is associated with first and second (and subsequent) births and whether this association differs across various indicators of socioeconomic status. Third, the KLIPS survey, which consists of a representative sample of households in urban areas, is not ideal to study urban–rural differences in the relationship between socioeconomic status and fertility behaviors. Studies based on other data that include various rural areas would be a valuable addition to the literature.

Despite these limitations, this study contributes to a comprehensive understanding of socioeconomic differentials in fertility behaviors in Korea by documenting the relationships between multiple indicators of socioeconomic status and parity-specific fertility. One of the main findings from this study is that men’s employment security and affordable housing are conducive to the transition to parenthood and subsequent births. Socioeconomic status driven by structural issues such as the real estate market, mortgage law, and employment relations cannot be easily addressed at the individual or household level. Therefore, large-scale policies with long-term investments (e.g., expanding affordable housing to newlyweds) may be more fruitful to reverse or at least halt the trend of falling fertility rates in Korea. This study’s findings on socioeconomic differentials in fertility have further important implications beyond Korean society, given that many other developed countries also face growing inequality – in particular employment precarity and rising housing prices, as well as fertility decline.

## Figures and Tables

**Table 1:** Sample characteristics, by birth order

*Variable*	First birth		Second birth	
	*M/%*	*SD*	*M/%*	*SD*
Births	22.77		12.69	
Time since first marriage/birth	3.55	1.06	7.08	6.76
Wife’s age	34.64	7.45	35.58	6.45
Husband’s age	37.43	8.79	38.08	6.98
Sex of first child (1=male)^1^	N/A		57.65	
Region				
Seoul	23.86		21.73	
Metropolitan cities	26.12		28.04	
Other areas	50.02		50.23	
***Wife’s socioeconomic status***				
Educational attainment				
Less than high school	14.71		9.22	
High school	33.83		38.93	
Some college / junior college	21.03		24.25	
University or more	30.43		27.60	
Employment status				
Standard employment	37.27		26.24	
Nonstandard employment	7.27		6.78	
Self-employment	13.67		10.73	
Nonemployment	40.79		56.25	
***Husband’s socioeconomic status***				
Educational attainment				
Less than high school	12.54		8.20	
High school	26.56		33.22	
Some college / junior college	23.12		21.54	
University or more	37.79		37.04	
Employment status				
Standard employment	65.39		65.53	
Nonstandard employment	6.53		6.69	
Self-employment	20.64		21.74	
Nonemployment	7.44		6.03	
***Household socioeconomic status***				
Annual income (logged)^2^	3.39	0.80	3.43	0.73
Homeownership				
Homeowner	38.75		48.34	
*Jeonse* (key money deposit)	42.79		37.69	
*Weolse* (monthly rent)	11.97		10.03	
Other	6.49		3.94	
*Person-year observations*	2,297		7,938	
*Number of individuals*	889		1,992	

1 Dichotomous variable

2 Annual household income is measured in 1,000,000 KRW, approximately 830 US dollars.

**Table 2:** Results from discrete-time hazard models predicting the transition to first birth

Variable	M1	M2	M3	M4	M5	M6	M7
Wife’s age	−0.083 [−0.127, −0.040]	−0.074 [−0.116, −0.033]	−0.070 [−0.110, −0.029]	−0.072 [−0.113, −0.031]	−0.076 [−0.116, −0.035]	−0.075 [−0.116, −0.034]	−0.076 [−0.120, −0.031]
Husband’s age	−0.037 [−.077, 0.004]	−0.048 [−0.088, −0.009]	−0.060 [−0.098, −0.021]	−0.054 [−0.093, −0.015]	−0.051 [−0.089, −0.013]	−0.052 [−0.091, −0.013]	−0.052 [−0.095, −0.010]
Region							
Seoul (omitted)							
Metropolitan cities	0.178 [−.137, 0.493]	0.151 [−0.163, 0.464]	0.111 [−0.200, 0.422]	0.131 [−0.180, 0.442]	0.116 [−0.194, 0.426]	0.103 [−0.209, 0.416]	0.190 [−0.131, 0.511]
Other areas	0.020 [−.261, 0.301]	0.004 [−0.277, 0.285]	−0.034 [−0.314, 0.246]	−0.019 [−0.299, 0.261]	−0.018 [−0.297, 0.261]	−0.022 [−0.304, 0.260]	−0.008 [−0.295, 0.279]
Time since first marriage							
0–1 year (omitted)							
2–3 years	−0.318 [−0.568, −0.069]	−0.347 [−0.595, −0.098]	−0.355 [−0.604, −0.106]	−0.338 [−0.587, −0.089]	−0.353 [−0.602, −0.103]	−0.357 [−0.606, −0.108]	−0.333 [−0.589, −0.077]
4–5 years	−0.686 [−1.056, −0.317]	−0.728 [−1.094, −0.362]	−0.783 [−1.151, −0.415]	−0.744 [−1.111, −0.378]	−0.748 [−1.114, −0.382]	−0.743 [−1.109, −0.376]	−0.737 [−1.113, −0.361]
6+ years	−1.613 [−2.135, −1.092]	−1.713 [−2.231, −1.195]	−1.805 [−2.323, −1.286]	−1.791 [−2.311, −1.271]	−1.801 [−2.320, −1.281]	−1.816 [−2.341, −1.291]	−1.620 [−2.144, −1.096]
Wife’s educational attainment							
Less than high school	−1.575 [−2.625, −0.525]						−1.562 [−2.656, −0.469]
High school (omitted)							
Some college / junior college	0.326 [0.041, 0.612]						0.320 [−0.003, 0.642]
University or more	0.361 [0.097, 0.624]						0.343 [−0.011, 0.696]
Husband’s educational attainment							
Less than high school		−0.643 [−1.438, 0.153]					−0.158 [−1.024, 0.709]
High school (omitted)							
Some college / junior college		0.167 [−0.143, 0.476]					−0.014 [−0.359, 0.331]
University or more		0.329 [0.048, 0.609]					0.068 [−0.300, 0.437]
Wife’s employment status							
Non-employment (omitted)							
Standard employment			−0.435 [−0.674, −0.196]				−0.477 [−0.724, −0.230]
Nonstandard employment			−0.526 [−1.000, −0.052]				−0.562 [−1.048, −0.076]
Self-employment			−0.434 [−0.869, 0.001]				−0.451 [−0.908, 0.005]
Husband’s employment status							
Non-employment (omitted)							
Standard employment				0.899 [0.345, 1.453]			0.830 [0.263, 1.397]
Nonstandard employment				0.739 [−0.026, 1.504]			0.927 [0.124, 1.730]
Self-employment				0.917 [0.296, 1.539]			0.957 [0.317, 1.598]
Annual household income (logged)^1^					0.046 [−0.097, 0.189]		−0.036 [−0.194, 0.123]
Homeownership							
Homeowner (omitted)							
*Jeonse* (key money)						−0.187 [−0.438, 0.063]	−0.171 [−0.426, 0.084]
*Weolse* (monthly rent)						−0.556 [−0.974, −0.138]	−0.419 [−0.853, 0.015]
Other						−0.071 [−0.534, 0.393]	−0.107 [−0.581, 0.368]
Constant	2.998 [1.984, 4.011]	3.137 [2.136, 4.139]	3.852 [2.849, 4.854]	2.602 [1.500, 3.705]	3.322 [2.299, 4.346]	3.647 [2.617, 4.678]	2.989 [1.741, 4.237]
Person-year observations	2,297	2,297	2,297	2,297	2,297	2,297	2,297

1 Annual household income is measured in 1,000,000 KRW, approximately 830 USD.

95% confidence intervals in brackets.

**Table 3:** Results from discrete-time hazard models predicting the transition to second birth

Variable	M1	M2	M3	M4	M5	M6	M7
Wife’s age	−0.102 [−0.130, −0.074]	−0.105 [−0.133, −0.077]	−0.097 [−0.124, −0.069]	−0.100 [−0.128, −0.073]	−0.105 [−0.133, −0.077]	−0.101 [−0.129, −0.073]	−0.107 [−0.135, −0.078]
Husband’s age	−0.012 [−.039, 0.014]	−0.014 [−0.040, 0.013]	−0.017 [−0.042, 0.009]	−0.014 [−.040, 0.012]	−0.017 [−.042, 0.009]	−0.015 [−.041, 0.010]	−0.016 [−0.043, 0.011]
Sex of first child (1=male)	−0.141 [−.283, 0.001]	−0.142 [−0.284, 0.000]	−0.151 [−0.293, −0.009]	−0.150 [−0.292, −0.008]	−0.150 [−0.292, −0.008]	−0.157 [−0.299, −0.015]	−0.147 [−0.290, −0.004]
Region							
Seoul (omitted)							
Metropolitan cities	0.066 [−.142, 0.274]	0.088 [−0.122, 0.298]	0.050 [−0.159, 0.258]	0.060 [−.148, 0.268]	0.070 [−.138, 0.278]	0.051 [−.158, 0.261]	0.077 [−0.135, 0.289]
Other areas	−0.009 [−.201, 0.182]	0.002 [−0.191, 0.194]	−0.025 [−0.216, 0.166]	−0.012 [−.203, 0.179]	−0.007 [−.197, 0.184]	−0.015 [−.207, 0.177]	−0.013 [−0.207, 0.181]
Time since first marriage							
0–1 year (omitted)							
2–3 years	0.433 [0.271, 0.595]	0.441 [0.279, 0.604]	0.440 [0.277, 0.602]	0.432 [0.270, 0.594]	0.430 [0.268, 0.593]	0.430 [0.267, 0.592]	0.453 [0.289, 0.617]
4–5 years	−0.202 [−.437, 0.034]	−0.180 [−0.416, 0.056]	−0.198 [−0.433, 0.037]	−0.212 [−.446, 0.021]	−0.208 [−.442, 0.025]	−0.213 [−.447, 0.021]	−0.148 [−0.387, 0.091]
6+ years	−1.738 [−2.096, −1.380]	−1.708 [−2.066, −1.350]	−1.779 [−2.133, −1.425]	−1.789 [−2.141, −1.437]	−1.762 [−2.115, −1.408]	−1.784 [−2.138, −1.430]	−1.647 [−2.009, −1.285]
Wife’s educational attainment							
Less than high school	−0.532 [−.088, 0.023]						−0.320 [−0.928, 0.289]
High school (omitted)							
Some college / junior college	0.090 [−.087, 0.267]						0.005 [−0.188, 0.198]
University or more	0.094 [−.084, 0.272]						−0.057 [−0.282, 0.169]
Husband’s educational attainment							
Less than high school		−0.391 [−0.880, 0.099]					−0.166 [−0.704, 0.372]
High school (omitted)							
Some college / junior college		0.086 [−0.108, 0.279]					0.040 [−0.167, 0.248]
University or more		0.197 [0.021, 0.373]					0.181 [−0.040, 0.402]
Wife’s employment status							
Non-employment (omitted)							
Standard employment			−0.153 [−0.324, 0.018]				−0.228 [−0.410, −0.047]
Nonstandard employment			−0.071 [−0.439, 0.297]				−0.056 [−0.428, 0.317]
Self-employment			−0.229 [−0.533, 0.075]				−0.223 [−0.538, 0.093]
Husband’s employment status							
Non-employment (omitted)							
Standard employment				0.375 [0.010, 0.739]			0.260 [−0.116, 0.636]
Nonstandard employment				0.109 [−.386, 0.605]			0.114 [−0.386, 0.615]
Self-employment				0.364 [−.034, 0.763]			0.318 [−0.094, 0.730]
Annual household income (logged)^2^					0.123 [0.017, 0.229]		0.095 [−0.031, 0.221]
Homeownership							
Homeowner (omitted)							
*Jeonse* (key money)						−0.047 [−.203, 0.109]	−0.020 [−0.178, 0.138]
*Weolse* (monthly rent)						−0.318 [−0.603, −0.034]	−0.207 [−0.500, 0.086]
Other						−0.019 [−.338, 0.299]	0.016 [−0.305, 0.336]
Constant	2.148 [1.459, 2.836]	2.211 [1.521, 2.900]	2.246 [1.567, 2.924]	1.875 [1.117, 2.633]	2.019 [1.315, 2.722]	2.335 [1.617, 3.052]	1.916 [1.094, 2.739]
Person-year observations	7,938	7,938	7,938	7,938	7,938	7,938	7,938

1 Dichotomous variable

2 Annual household income is measured in 1,000,000 KRW, approximately 830 USD.

95% confidence intervals in brackets.

**Figure:**
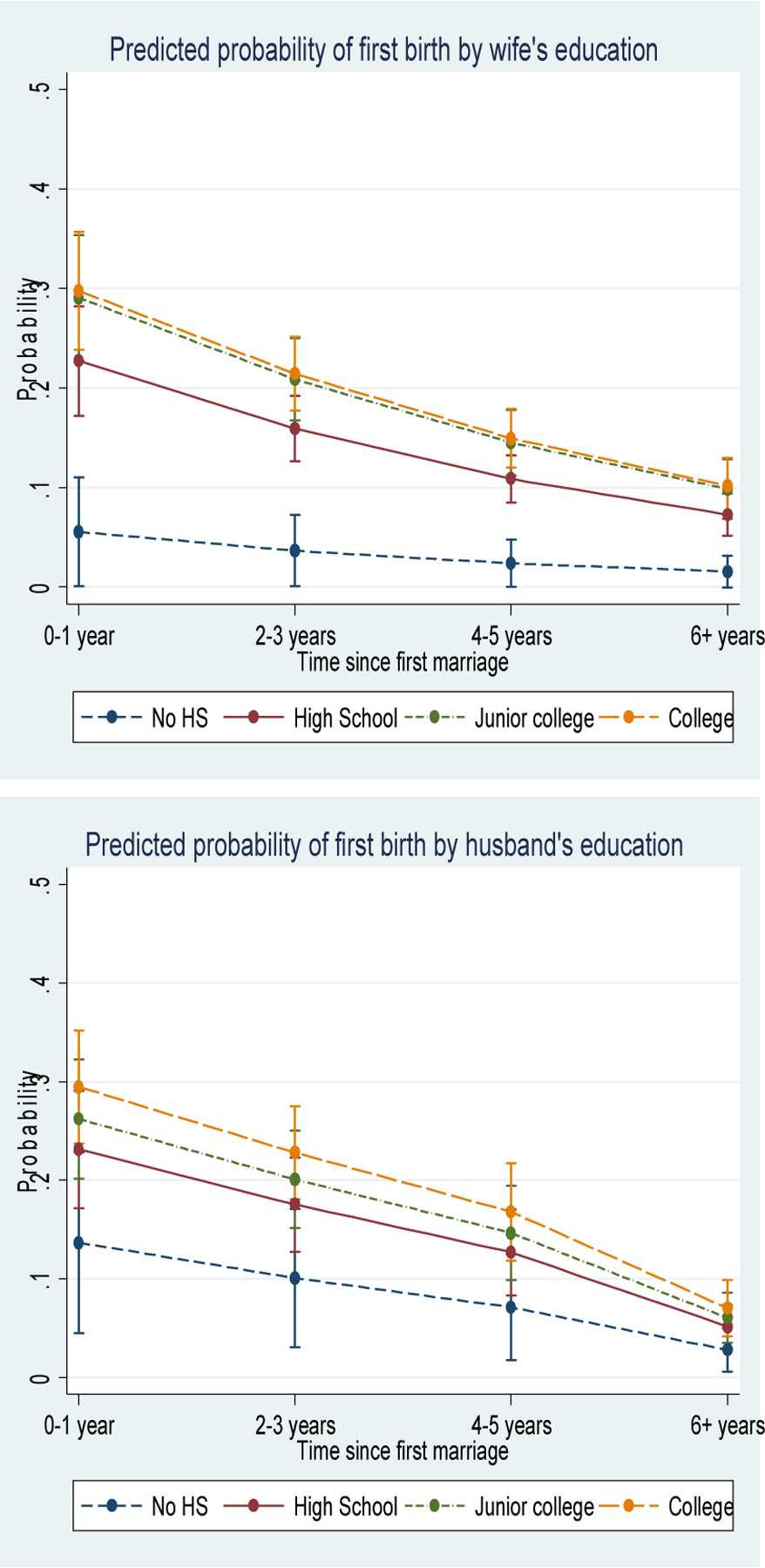
*Notes*: The figures illustrate probabilities of women having a first birth at each duration with 95% confidence intervals while holding all covariates at their mean across different levels of education. All models include wife’s age, husband’s age, place of residence, and time since first marriage in the form of splines of duration, in addition to wife’s education (the top figure, Model 1, Table 2) and husband’s education (the bottom figure, Model 2, Table 2).

**Figure:**
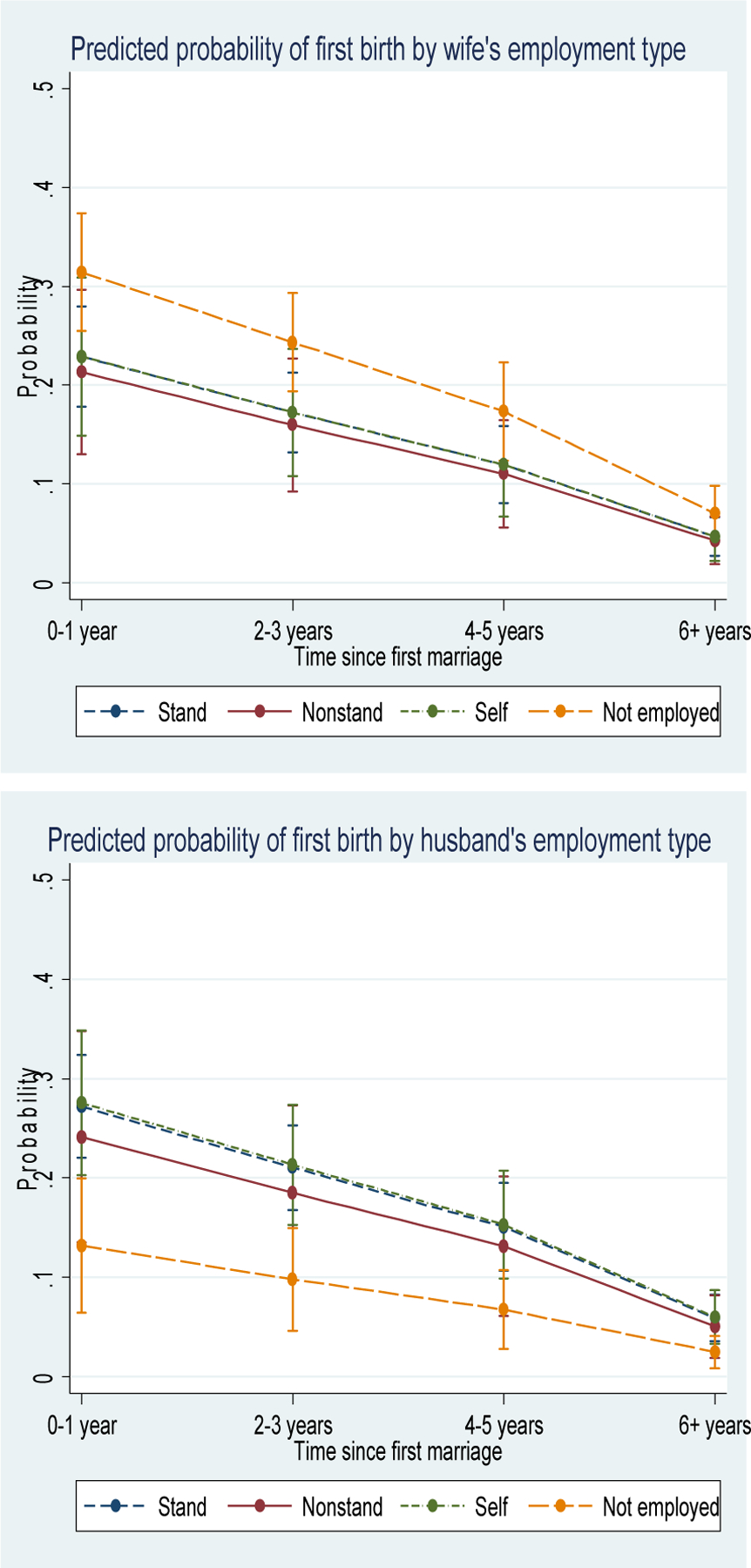
*Notes*: The figures illustrate probabilities of women having a first birth at each duration with 95% confidence intervals while holding all covariates at their mean across different employment types. All models include wife’s age, husband’s age, place of residence, and time since first marriage in the form of splines of duration, in addition to wife’s employment type (the top figure, Model 3, Table 2) and husband’s employment type (the bottom figure, Model 4, Table 2).

**Figure:**
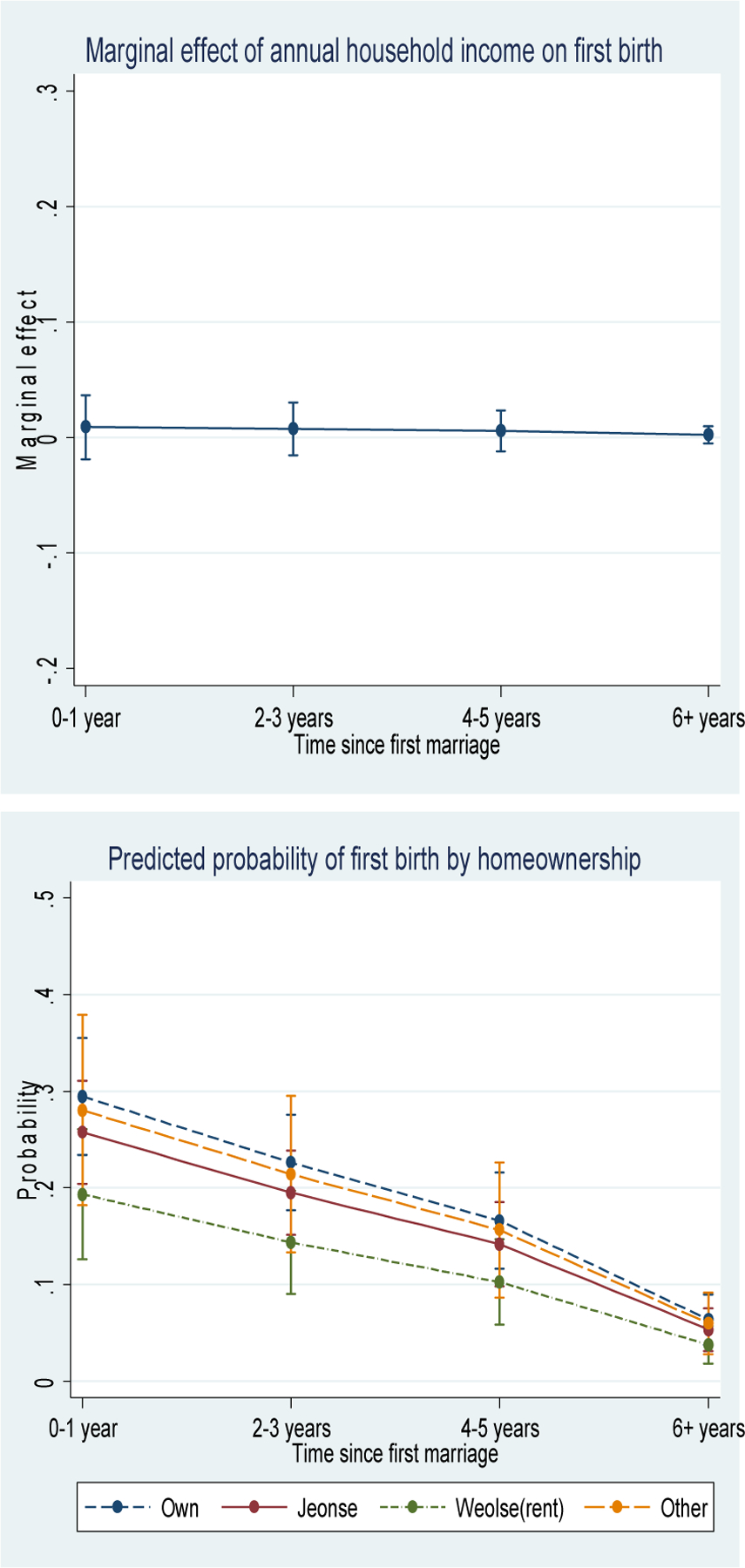
*Notes*: The figures illustrate probabilities of women having a first birth at each duration with 95% confidence intervals while holding all covariates at their mean across household income (the top figure) and homeownership status (the bottom figure). The figure for household income shows a marginal effect of an additional 1,000,000 KRW (approximately 850 USD) in household income (annual, logged) on first births across duration. All models include wife’s age, husband’s age, place of residence, and time since first marriage in the form of splines of duration, in addition to annual household income (the top figure, Model 5, Table 2) and homeownership status (the bottom figure, Model 6, Table 2).

**Figure:**
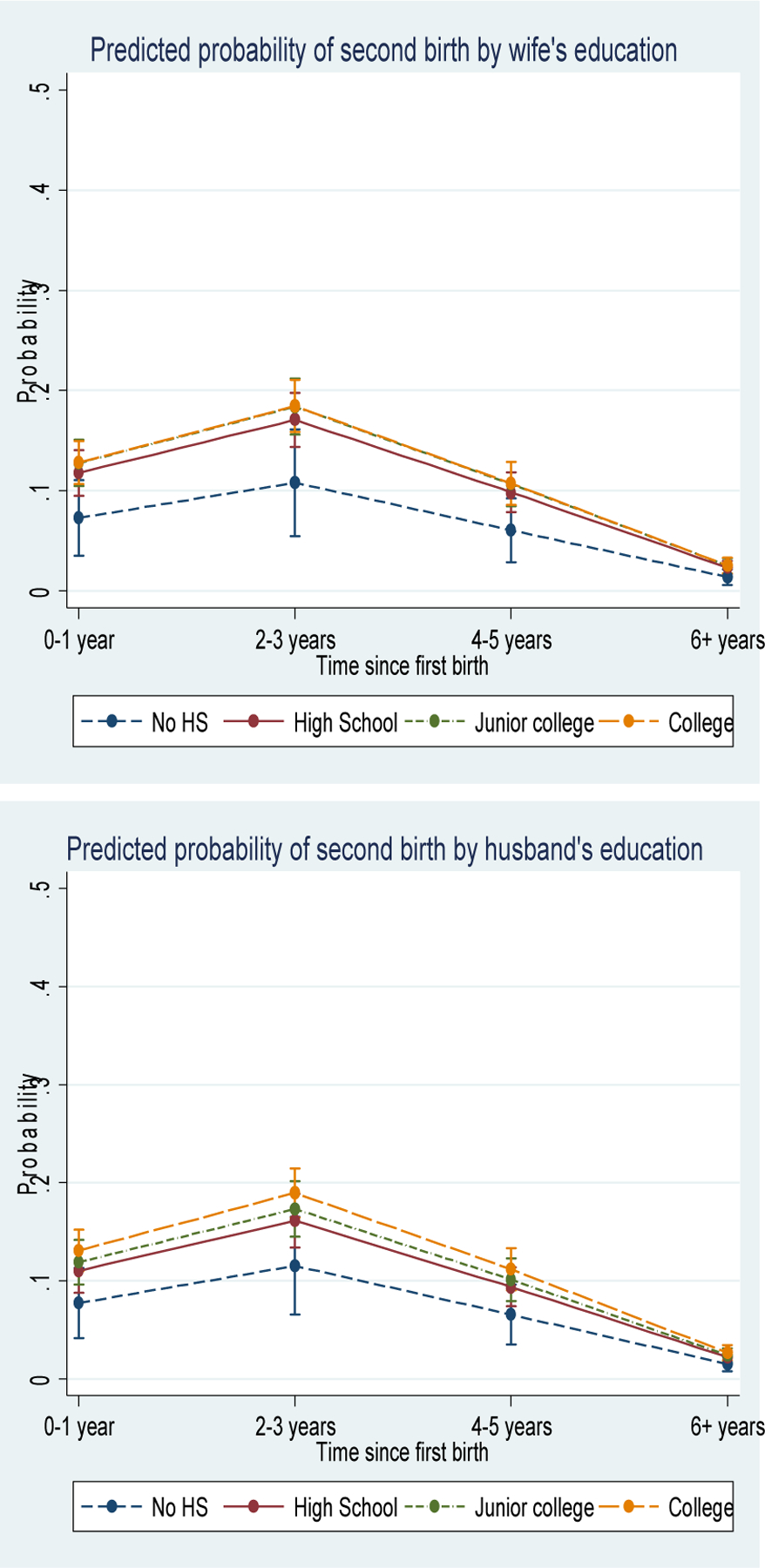
*Notes*: The figures illustrate probabilities of women having a second birth at each duration with 95% confidence intervals while holding all covariates at their mean across different levels of education. All models include wife’s age, husband’s age, the sex of first child, place of residence, and time since first birth in the form of splines of duration, in addition to wife’s education (the top figure, Model 1, Table 3) and husband’s education (the bottom figure, Model 2, Table 3).

**Figure:**
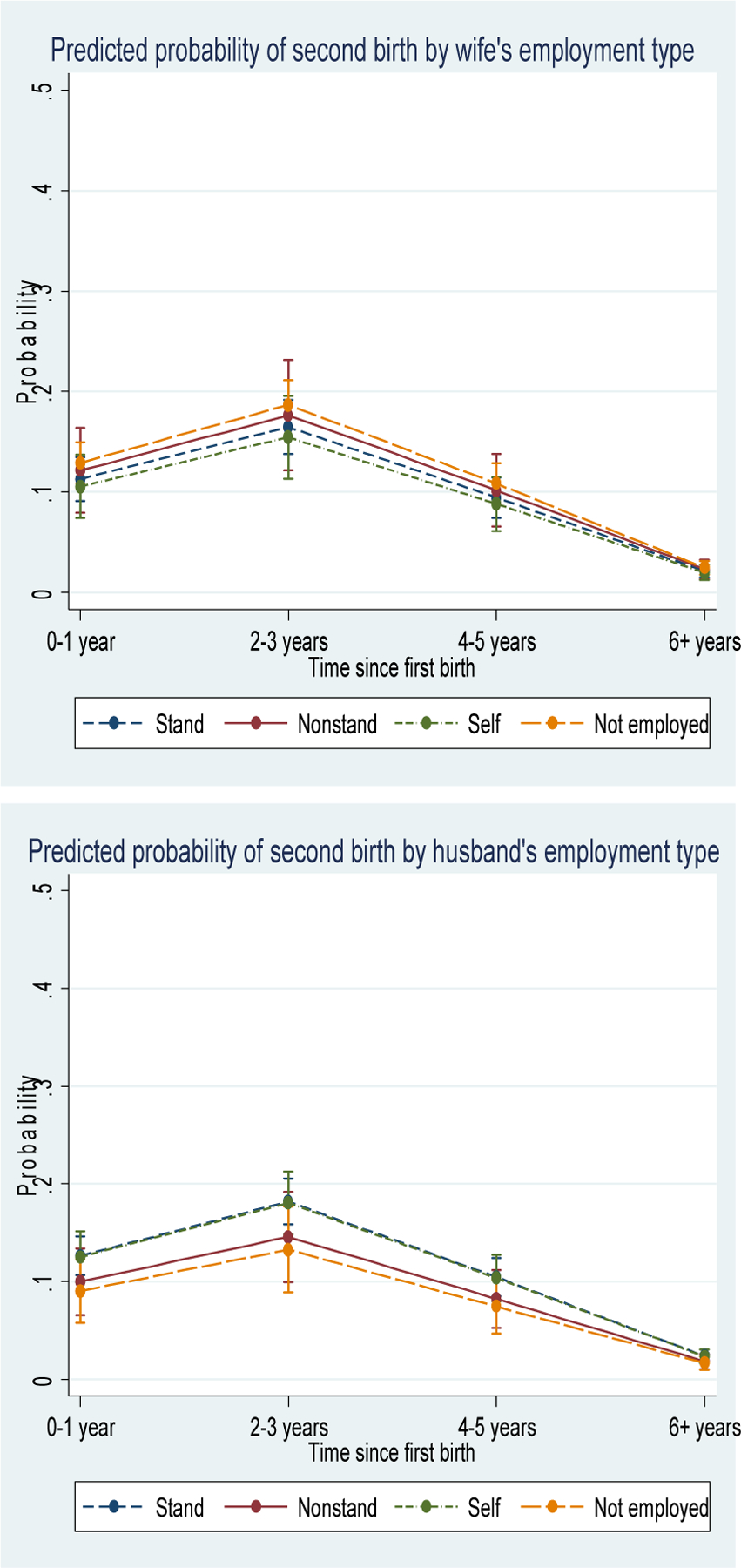
*Notes*: The figures illustrate probabilities of women having a second birth at each duration with 95% confidence intervals while holding all covariates at their mean across different employment types. All models include wife’s age, husband’s age, the sex of first child, place of residence, and time since first birth in the form of splines of duration, in addition to wife’s employment type (the top figure, Model 3, Table 3) and husband’s employment type (the bottom figure, Model 4, Table 3).

**Figure:**
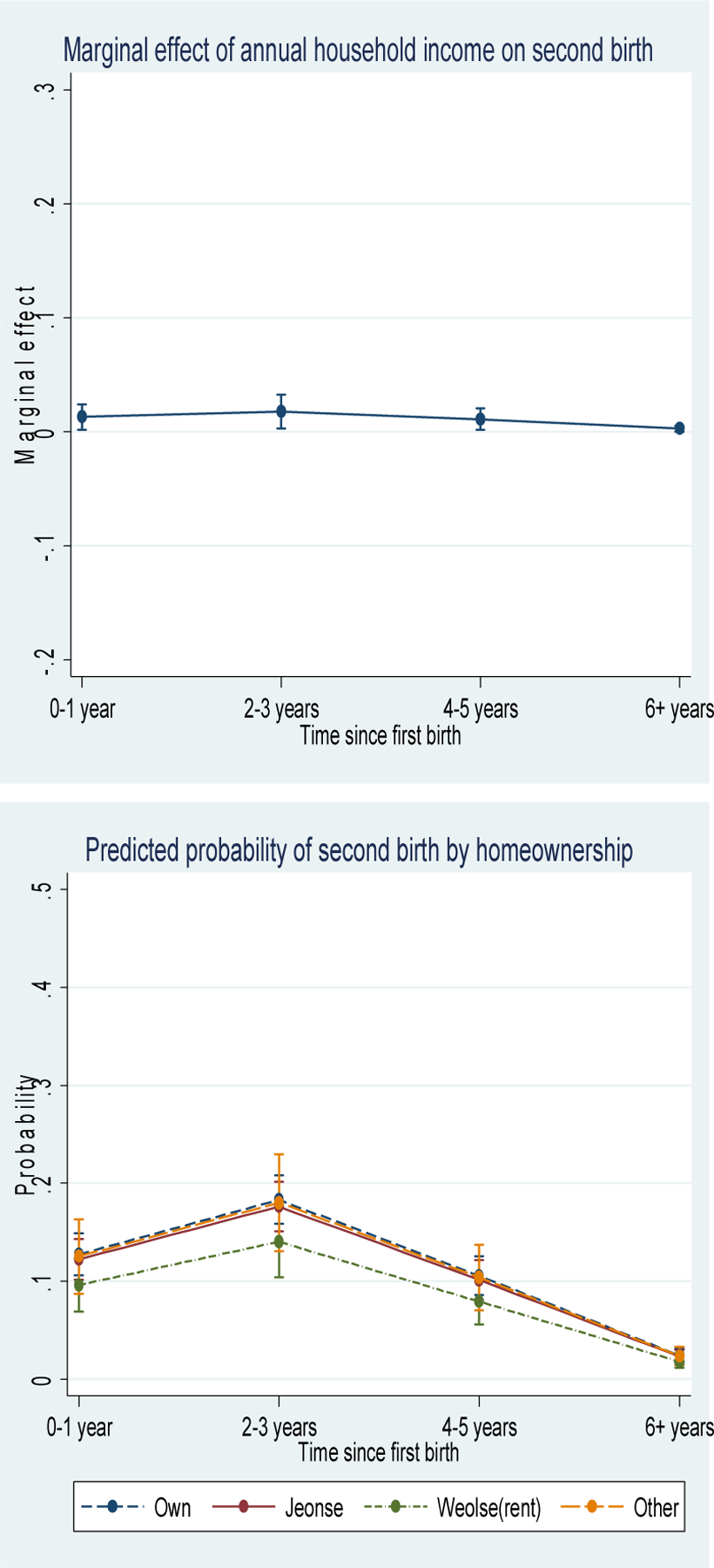
*Notes*: The figures illustrate probabilities of women having a second birth at each duration with 95% confidence intervals while holding all covariates at their mean across household income (the top figure) and homeownership status (the bottom figure). The figure for household income shows a marginal effect of an additional 1,000,000 KRW (approximately 850 USD) in household income (annual, logged) on second births across duration. All models include wife’s age, husband’s age, the sex of first child, place of residence, and time since first birth in the form of splines of duration, in addition to annual household income (the top figure, Model 5, Table 3) and homeownership status (the bottom figure, Model 6, Table 3).

## Footnotes

2 As the rate of nonmarital births is very low in Korea, the level of fertility is mostly determined by marital fertility. For this reason, the term ‘fertility’ is used throughout the paper without differentiating marital fertility from nonmarital fertility. Analyses in the present study were restricted to those in a first marriage (see the Data and Methods section).

3 It is worth noting that many scholars in the United States and Europe classify self-employment as a form of nonstandard employment arrangement (e.g., Kalleberg 2000), whereas in South Korea, government statistics separate self-employment (classified as nonwaged work) from other nonstandard employment (classified as waged work).

4 Ancillary analyses were conducted in order to see whether there were systematic patterns among missing cases. While those with missing information on one variable (e.g., time since the first birth) are likely to have missing information on another (e.g., the gender of first child or annual household income), a notable pattern was not identified. In addition, models were estimated after imputing missing information (e.g., annual household income), but the substantial conclusions remained the same.

5 Among those who were at risk of childbirth during the observation period, the average duration between marriage and the first birth is 17 months. Similarly, the average time period in the analytic sample between the first birth and the second birth is 36 months. These patterns are consistent with the findings from other data that in Korea married couples experience first births (as well as any subsequent births) within a relatively short period of time (Statistics Korea 2019). In light of the timing of first and second births within marriage and the structure of the data (i.e., annual data and a person-year observation unit of analysis), the baseline hazard is defined by splines of duration.

6 As the observational period of the KLIPS used in the study covers nearly two decades and the KLIPS also includes different birth cohorts, supplementary analyses were conducted to examine whether the patterns of socioeconomic differences in first and second births differ across cohorts (i.e., those born before 1970, between 1971 and 1980, and after 1981) and between different periods (1998–2009 and 2010–2017). Results show that there are no notable cohort and period effects in either first or second births, except that the later period (2010–2017) is more negatively associated with the likelihood of having first births than the earlier period, reflecting the continuous very low fertility regime observed in the past couple of decades. In addition, in the later period the positive association between husband’s standard job and self-employment and the transition to first birth becomes stronger; however, the general pattern remains the same. The growing importance to first births of the husband’s employment conditions in the later period is consistent with recent evidence of the positive association between socioeconomic status and fertility level in the context of very low fertility after the economic crisis (Kim 2013).

7 In the case of annual household income, which is a continuous variable, a marginal effect is presented instead of predicted probabilities. In order to illustrate the magnitude of the difference in predicted probabilities across different levels of socioeconomic status, the predicted probability of a household with an annual income of 51,000,000 KRW (the average household income reported in 2017, Statistics Korea 2017) having a first birth is 0.229 and having a second birth is 0.129. This information can, for example, be used to compare the difference of 0.25 in predicted probabilities of a first birth between women without high school education and women with a college degree (the top figure, Appendix A) with reference to the predicted probability of 0.229 for a household with average annual income (51,000,000 KRW in 2017) having a first birth.

8 As noted in the Results section, some of the associations between a specific indicator and childbirths change in the presence of other SES indicators (Tables 2 and 3). The results need to be interpreted cautiously while taking this into consideration.

9 It is worth noting that the negative association between *Weolse* (monthly rent) and the first/second childbirth attenuates in the full model that includes all indicators of socioeconomic status (Model 7, Tables 2 and 3).
